# Metastatic Seeding of Abdominal Wall After Drainage of Perforated Colorectal Cancer in a Presumed Complicated Diverticular Disease

**DOI:** 10.7759/cureus.41469

**Published:** 2023-07-06

**Authors:** David P Le

**Affiliations:** 1 Internal Medicine, University of South Alabama, Mobile, USA

**Keywords:** perforated colon cancer, percutaneous abscess drainage, metastatic abdominal wall tumor, diverticular abscess, colorectal cancer, complicated diverticular disease

## Abstract

A 37-year-old male presented multiple times for abdominal pain with a persistent diverticular abscess on imaging that was managed previously with antibiotics and percutaneous drainages. Due to unrelenting abdominal pain and multiple presentations of unresolved acute complicated diverticulitis, the patient underwent an exploratory laparotomy. A colonic mass was discovered, and the patient had a colonic resection. Pathology revealed invasive transverse colonic adenocarcinoma with perforation and involvement of the stomach. Imaging showed no distant metastatic disease and chemotherapy was started. Months after treatment, the patient developed skin lesions and a palpable mass at the previous drain site. Biopsy showed metastatic adenocarcinoma consistent with colonic origin. Colonic adenocarcinoma with metastasis to the abdominal wall after drainage of presumed diverticular abscess is rare. Clinicians should consider malignancy when a patient has a recurrent diverticular abscess that has failed medical management and multiple drainages. Clinicians should remain vigilant of the risk of seeding colonic adenocarcinoma to the abdominal wall when repeated drainage is performed.

## Introduction

Diverticular disease is an uncommon colonic disease estimated to be as low as 5% in ages 40 years or younger [1]. Diverticular complications such as perforation, abscess, or fistula formation can occur. Patients 40 years or younger have a more aggressive form of diverticular disease and have a higher likelihood of complications requiring surgical intervention [1]. Rarely, colon cancer can clinically and radiologically mimic an acute complicated diverticulitis (ACD) that has perforated or formed an abscess. Severe presentation of complicated diverticular disease is typically managed by expedient hospitalization, percutaneous image-guided drainage, and parenteral antibiotics [2]. A computed tomography (CT) scan is the gold standard and imaging modality of choice to diagnose diverticular disease from other intra-abdominal pathologies. Other complications of diverticular disease can be seen on CT imaging such as diverticulitis, abscess, phlegmon, fistula, or malignancy. Patients with features of complicated acute diverticulitis on imaging may actually represent an underlying colon cancer [3].

Colorectal cancer (CRC) is one of the most common cancers worldwide. Common initial presenting symptoms of CRC include abdominal pain, palpable abdominal mass, change in bowel habits, rectal bleeding, unexplained anemia, and weight loss. Abscess formation and perforation are less common presentations of CRC. A well-contained perforation and local abscess formation of CRC can resemble complicated diverticular disease both in terms of symptomatology and radiographic findings.

A combination of antibiotics, bowel rest, and drainage is the usual approach to manage diverticular disease. However, in patients with unresolved and persistent diverticular disease, it is imperative for clinicians to consider perforated colonic carcinoma before draining the fluid collection due to possible risk of metastatic tumor seeding to the abdominal wall at the drainage site.

## Case presentation

**Figure 1:**
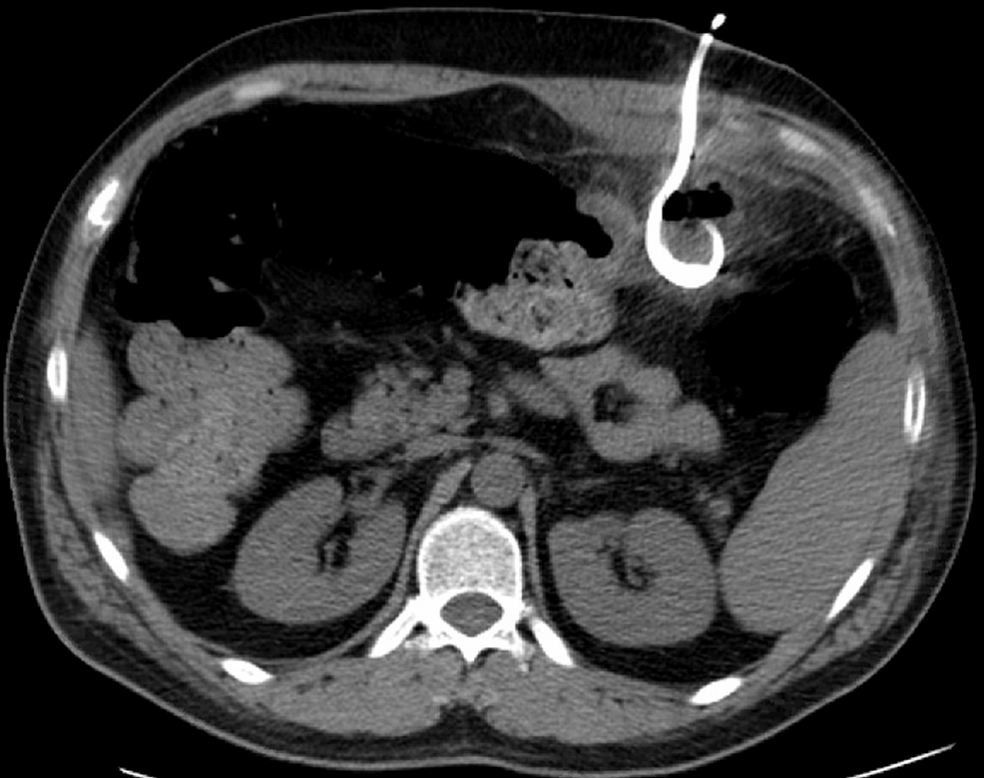
Image-guided CT peritoneal catheter drainage placement within the fluid collection. CT, computerized tomography

A 37-year-old male with a past medical history of iron deficiency anemia and diverticulitis with diverticular abscess and fistulization presented for a third time to the emergency department for persistent left upper quadrant abdominal pain and constipation for the past week. The patient was previously hospitalized for management of his diverticular abscess with intravenous antibiotics and image-guided percutaneous drainages (Figure 1). He was discharged recently after his abdominal pain improved but did not follow up for his scheduled outpatient colonoscopy after each episode of diverticulitis since he would shortly return to the hospital for intractable abdominal pain. He had no family history of cancer, diabetes, hypertension, or bleeding/coagulopathy disorders. He denies smoking, alcohol, and illicit drug use. The patient had a gradual but constant, dull achy non-radiating left upper abdominal pain, nausea, and decreased ability for oral intake. He unintentionally lost 20 pounds of weight over the past six months. The patient denied fever, chills, diarrhea, hematochezia, or melena. The patient was afebrile and vitally stable. On physical exam, the patient’s abdomen was distended with tenderness to palpation in the left upper quadrant.

Patient’s laboratory results showed a leukocytosis, iron deficiency anemia, and normal carcinoembryonic antigen (Table 1).

**Table 1:** Summary of significant laboratory findings

Laboratory Tests	Values	Normal Values
Hemoglobin	11.1 g/dL	13.5-17.5
MCV	69 fL	80-100
Ferritin	3 ng/mL	48-420
Iron	17 mcg/dL	65-175
Iron Binding Capacity	431 mcg/dL	250-450
White blood cell count	18.6 x10^3 / mcL	4.3-10
Absolute neutrophil count	16.52 x 10^3 / mcL	1.8-7.3
Carcinoembryonic antigen	< 0.5 ng/mL	1.0-4.1

**Figure 2:**
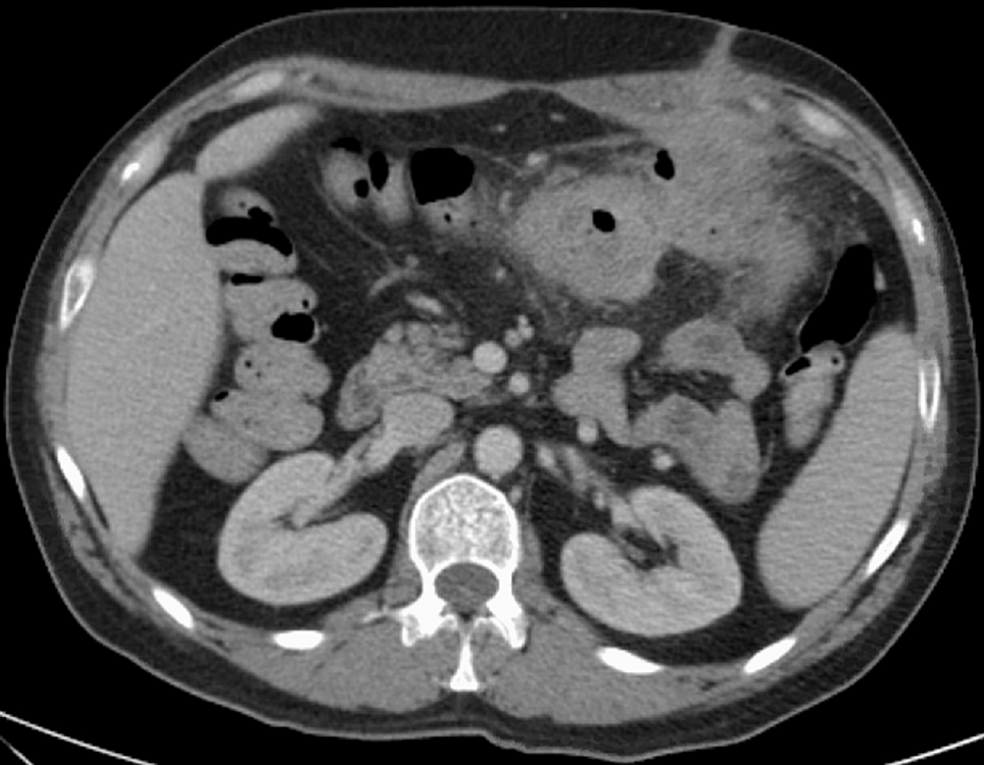
Redemonstrated mesenteric fat stranding with overall unchanged ill-defined air-fluid collection around transverse colon at the previous site of diverticular abscess. Prominent left upper quadrant mesenteric lymph nodes. Soft tissue density in the abdominal wall at the previous drain site.

Contrast-enhanced abdominal CT revealed redemonstration of the mesenteric fat stranding with unchanged air-fluid collection around the transverse colon, with prominent left upper quadrant mesenteric lymph nodes, and a new soft tissue density in the abdominal wall at the site of previous drain site (Figure 2).

**Figure 3:**
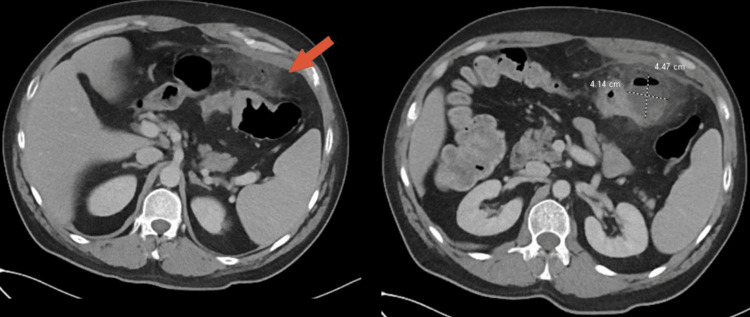
CT abdomen and pelvis with contrast demonstrating evidence of perforated diverticulitis with abscess formation measuring 4.14 x 4.47 x 5.12 cm. Discrete hypodense collection in the left upper quadrant with evidence of stranding and inflammatory changes adjacent to the transverse and splenic flexure (arrow). CT, computerized tomography

With signs and symptoms of possible bowel obstruction, intractable abdominal pain, previous failed medical management with percutaneous drainage and antibiotics, surgical intervention with exploratory laparotomy and colonic resection was planned. There was a concern for underlying malignancy given features of perforated diverticulitis with fluid collection and inflammatory changes near the splenic flexure and transverse colon (Figure 3). However, malignancy was thought to be unusual and rare given the patient’s young age and no other typical colonic cancer symptoms such as hematochezia and exophytic mass.

**Figure 4:**
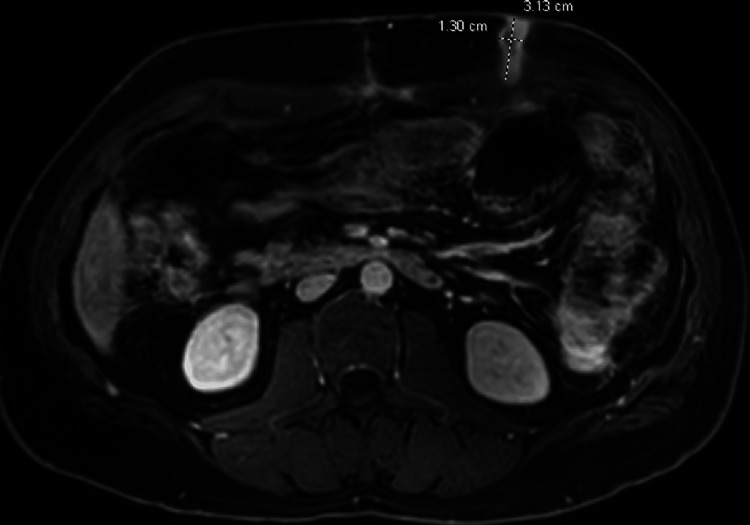
MRI abdomen with and without contrast showing enhancement of subcutaneous soft tissues around site of prior abdominal drain. Also represent normal enhancement of fibrous/scar tissue, direct seeding of cutaneous/subcutaneous site cannot be completely excluded. MRI, Magnetic resonance imaging

The patient ultimately underwent exploratory laparotomy, with the discovery of a colonic mass. Patient had a right colectomy, gastric wedge resection, and partial omentectomy. Pathology revealed a 11-cm moderately differentiated invasive transverse colonic adenocarcinoma with perforation and involvement of the stomach. The lymph node and malignancy resection margin were negative and final tumor-node-metastasis (TNM) staged as pT4bN0. He was subsequently started on FOLFOX (fluorouracil, folinic acid, and oxaliplatin) for his colon cancer. After three cycles of chemotherapy, the patient noticed new blisters and skin lesions around the prior drain site of his abscess. He underwent a skin biopsy which showed metastatic disease to the abdominal wall with primary colonic origin. Magnetic resonance imaging (MRI) showed the progression of metastatic abdominal wall tumor with local invasion and the development of a palpable mass at the previous drain site (Figure 4).

The patient underwent radical resection of his abdominal wall metastasis, pathology revealed moderately differentiated adenocarcinoma, extending to less than 1 mm from the deep peritoneal margin. With discussion between multidisciplinary team involving medical oncology, colorectal surgery, and surgical oncology patient had a radical resection of his abdominal wall mass and the composite mesh was placed. The peritoneum was accessed, and no involvement of the bowel was seen. The patient was restarted on his chemotherapy regimen and has continued to do well on his subsequent follow-up visits.

## Discussion

In the United States, acute diverticulitis is one of the most common gastrointestinal illnesses that is a cause of multiple hospital admissions [4]. Acute diverticulitis in the United States is also one of the leading indications for elective colonic resection [4]. Acute diverticulitis is inflammation in and adjacent to a diverticulum or sac-like protrusion of the colonic wall. ACD is defined as diverticulitis with one complication such as bowel obstruction, abscess, fistula, or perforation. A patient with acute diverticulitis usually presents with left lower quadrant pain, abdominal tenderness, and leukocytosis on laboratory investigation. An abdominal and pelvic CT scan with contrast can help distinguish between uncomplicated and complicated diverticular disease. Uncomplicated diverticulitis may involve only local peri-diverticular inflammation with CT finding of localized bowel wall thickening and pericolonic fat stranding. For acute complications of diverticular disease on CT, abscesses are visualized as fluid collections with adjacent inflammatory changes in the surrounding areas.

ACD is treated in the inpatient setting with antibiotics and supportive care for colonic inflammation. Elective interventions or surgery are performed to treat the specific acute diverticulitis complication. For abscesses, percutaneous drainage is performed, or colonic resection for frank perforation. Up to 40% of patients with complicated acute diverticulitis have abscesses [5] and are managed with a combination of antibiotics and percutaneous drainage. Nonoperative approach for diverticular abscess with antibiotics, percutaneous drainage, or both has a high success rate; however, around 20% of patients still require surgery [6]. CT-guided drainages are performed for abscesses measuring four cm or greater [7]. A drainage catheter is placed through the anterior abdominal wall and left until output is minimal. Patients are regularly assessed daily and failure to improve with antibiotics and drainage should prompt for repeat imaging. Disease progression may show new abscess or perforation. For patients that do not improve after percutaneous drainage of a diverticular abscess within 48 hours or if a young patient with recurrent episodes of diverticulitis has unresolved symptoms within six weeks, referral to surgery should be considered.

Patients that are hospitalized for ACD usually have severe disease or multiple comorbidities requiring antibiotics and operative management [7]. Surgical management is indicated for ACD or at any point during hospitalization if the patient has intractable abdominal pain, develops signs of diffuse peritonitis, or has failed conservative medical management. The American Society of Colon and Rectal Surgeons (ASCRS) clinical guidelines recommend surgical intervention for a patient with at least one prior episode of complicated diverticulitis with complications [8]. Our patient had a localized perforation with a contained abscess in the pericolonic location that was treated with percutaneous image-guided drainage and intravenous antibiotic therapy multiple times. The patient initially responded well to medical treatment and was discharged, but subsequently developed recurrent symptoms of abdominal pain and bowel movement habit alterations that prompted close evaluation for surgery. Because symptoms may persist well after the patient’s episodes of treated diverticulitis, other gastrointestinal disorders such as irritable bowel syndrome, appendicitis, inflammatory bowel disease (IBD), colitis, and colon cancer can present similarly. The patient’s concerning symptoms of bowel obstruction, unresolved diverticular abscess, and iron deficiency anemia with CT evidence of proximal reactive lymph nodes were indicative of an underlying malignancy. Patients with colonic obstruction in the setting of acute diverticulitis should undergo surgical resection of the involved colonic segment because it is not possible to distinguish radiographically between a diverticular structure and a colonic carcinoma [9,10]. CT imaging alone should not be relied on to diagnose complicated diverticular disease from other intra-abdominal pathologies. On CT scan it may be difficult to exclude alternative conditions such as colon cancer due to many overlapping characteristics that is shared between complicated diverticular disease.

Risk factors for CRC include IBD, predisposed inheritable conditions (familial adenomatous polyposis, Lynch syndrome), African American descent, and diverticulitis. CRC can present in a variety of ways with either suspicious or emergent symptoms of obstruction, perforation, or an acute gastrointestinal bleed. The rare and unusual complications of colorectal carcinoma include bowel obstruction, abscess formation, and perforation. The predominance of left-sided colorectal carcinoma is due to the common occurrence of obstructing colonic lesions that are found in the smaller diameter of the left colon. Right-sided colonic lesions can present with symptoms of abdominal pain, palpable mass, or anemia. CT imaging can be used to detect bowel obstruction that can identify transitional zones and obstructing lesion on imaging that appears as irregular circumferential thickening of the colon [11].

Abscess formation with colon cancer is a rare complication in patients with colon cancer. The formation of an abscess can be due to perforation of the colon, fistula formation between the colon and adjacent structures, or direct tumor invasion [10]. Possible locations for abscess formation include the peritoneal cavity, retroperitoneum, abdominal wall, pararectal space, psoas muscle, thigh, and splenic and transverse flexure. Abscess-forming colon cancer that occurs in the abdominal lower quadrants or the pelvic cavity has been mistaken for other inflammatory conditions such as diverticulitis, appendicitis, and pelvic inflammatory disease [11].

Perforation and penetration of adjacent organs with abscess formation as an initial presentation of colorectal carcinoma are uncommon, with the incidence of perforation less than 10% [11]. Perforation of the peritoneal cavity locally can result in abscess or fistula formation. Perforation at the local site of the colon cancer is secondary to tumor necrosis or adjacent inflammation or can occur near the colon cancer due to increased pressure proximal to the tumor [12]. Symptoms of abdominal pain, bowel habit change, anemia, fever, leukocytosis, anorexia, diarrhea, and weight loss are commonly seen in perforated colon cancer. Colonic carcinoma can also perforate into the peritoneal cavity, which can be mistaken for diverticulitis especially when it occurs on the left side [11]. Atypical presentations for CRC with a localized contained perforation involving either the sigmoid or transverse colon may mimic diverticulitis in radiological appearance [11]. The differential for colonic perforation includes diverticulitis, trauma, ischemia, IBD, and malignancy. On CT imaging perforation of the colon may be demonstrated by a focal defect in the colon wall with fluid density abscess, free air, or stranding of the pericolic fat. However, the identification of severe pericolic inflammation and bowel wall thickening with a large abscess are not exclusive radiological findings. Clinical features and colonic wall thickening and inflammation are seen in both complicated diverticulitis and perforated colonic cancer.

Abdominal CT imaging or ultrasound is the most common technique to diagnose intra-abdominal pathologies. With abdominal CT, it has been reported that accurate diagnosis preoperatively of perforated colonic carcinoma is low [11]. Previous perforated colon cancer cases were either misdiagnosed as ruptured diverticulitis with abscess or fistula formation [13]. Differentiating between ACD and perforated colon cancer is difficult. If CRC with perforation or abscess is seen, CT findings can be non-specific. On CT imaging, colon cancer and inflammatory conditions can both show severe pericolic fat stranding adjacent to the thickened colon wall [14]. Asymmetric wall thickening, loss of stratified enhancement of the short segment of the colon, and abrupt transition from normal to abnormal bowel wall are characteristics of malignancy. In contrast, preservation of the thickened bowel wall is more likely a benign inflammatory process. On CT, the length of colonic inflammation may also help distinguish between a benign or a malignant process. For example, colonic carcinoma features include positive lymphadenopathy and usually less than five cm of colonic inflammation is involved [10]. However, inflammation involving more than ten cm of the colonic tract is more specific for diverticulitis [10].

Metastatic abdominal wall seeding after percutaneous drainage of a presumed complicated diverticular disease is rare. De Filippo et al. suggest that percutaneous drainage of abdominopelvic collections has been an alternative technique to surgery with lower morbidity and mortality rates [15]. Percutaneous drainages are commonly performed both in diverticular abscess complications as well as for intraperitoneal abscesses related to abdominal cancer. With striking similar radiological features compared to both ACD and complications of CRC, the approach to medically manage a large diverticular abscess is to perform drainage. For our case presentation, the patient himself did not suffer any complications during percutaneous drainage and the catheter was only placed with subsequent removal shortly after a few days. Quick reaccumulating intra-abdominal fluid should prompt the clinician to consider that underlying perforated colon cancer may be the case for an unresolved diverticular abscess. Each attempt of percutaneous drainage of a perforated colorectal carcinoma has a risk of tumor seeding to the abdominal wall. The patient can develop a subcutaneous palpable mass or new skin lesions at the previous puncture site of the percutaneous drainage catheter.

Abdominal wall drainage tract tumor seeding is incredibly rare and a few cases have been reported with gastric adenocarcinoma and metastatic abdominal wall implantation after drainage of intraabdominal abscess with a right colonic adenocarcinoma [16,17]. Tumor spread from percutaneous abdominal procedures is a rare, but possible complication. Seeding of metastatic tumor along drainage tracts have been reported in percutaneous transhepatic biliary drainages [18]. Soyer et al. proposed two ways in which tumors may seed to the abdominal wall after percutaneous drainages [17]. Tumor cells may potentially grow along the drainage catheter tract or multiple catheter exchanges and manipulations may lead to abdominal wall metastasis [19]. It is unclear the clinical significance of abdominal wall metastases after percutaneous drainage and how it may impact a patient’s survival rate.

Patients with diverticulitis should be screened for CRC after an acute episode of inflammation has resolved [20]. At six to eight weeks, patients with acute diverticulitis with a resolution of their symptoms should undergo a colonoscopy to screen for colon cancer unless if colonoscopy was performed within the previous year. It has been shown that colonoscopy detection of CRC is statistically higher after ACD compared to an episode of uncomplicated diverticulitis [20]. Patients with persistent or recurrent symptoms of the left lower quadrant patient change in bowel habits, and hematochezia may require surgical evaluation due to an underlying malignancy.

## Conclusions

It is important to consider malignancy when a patient presents multiple times with unresolved symptoms of ACD after several attempts with medical management with antibiotics and percutaneous drainages. This case highlights the importance of recognizing the radiological similarities between very different intra-abdominal pathologies. For example, the common finding of fat stranding around an inflamed structure seen on CT can be due to pancreatitis, appendicitis, cholecystitis, pyelonephritis, and diverticulitis. Other key radiological findings can help distinguish between ACD with abscess and perforated colonic cancer. Both clinical entities will show pericolic fat stranding, abnormal bowel wall thickening, and enhancement patterns on CT imaging, making colonic cancer and complicated diverticulitis a diagnostic challenge. Our case stresses the importance of considering the possible risk of colonic metastatic tumor seeding to the abdominal wall before performing multiple percutaneous abscess drainage in patients with presumed complicated diverticular disease that mimics perforated colonic cancer. The survival rate is worse with patients that have obstructive or perforated CRC; thus, it is crucial for clinicians to identify and reliably diagnose a perforated colon cancer to initiate timely management and improve clinical outcomes. Close follow-up with general surgery and gastroenterology is imperative after the acute episode of management of diverticulitis for screening for underlying CRC. More research will need to be conducted to identify distinguishing CT imaging features between perforated colonic cancer and ACD.
